# Supplementary material for: Anti-Ebola virus mAb 3A6 protects highly viremic animals from fatal outcome via binding GP(1,2) in a position elevated from the virion membrane
Source: Nat Commun. 2025 Feb 3;16:1293. doi: 10.1038/s41467-025-56452-2 (PMC11791206; doi:10.1038/s41467-025-56452-2)
Supplement: Supplementary file 1 — Supplementary Information [file 41467_2025_56452_MOESM1_ESM.pdf]

Supplementary Information

Anti-Ebola virus mAb 3A6 protects highly viremic animals from fatal outcome via binding GP(1,2) in a position elevated from the virion membrane

Kathryn M. Hastie[1], Zhe Li Salie[1,18,$], Zunlong Ke[2,3,15,$], Peter J. Halfmann[4], Lisa Evans DeWald[5], Sara McArdle[6], Ariadna Grinyó[7,20], Edgar Davidson[7], Sharon L. Schendel[1], Chitra Hariharan[1], Michael J. Norris[1,19], Xiaoying Yu[1,17], Chakravarthy Chennareddy[8], Xiaoli Xiong[2,16,], Megan Heinrich[9], Michael R. Holbrook[5], Benjamin Doranz[7], Ian Crozier[10], Yoshihiro Kawaoka[4,11,12,13], Luis M. Branco[9], Jens H. Kuhn[5], John A. G. Briggs[2,3,*], Gabriella Worwa[5,*], Carl W. Davis[8,*], Rafi Ahmed[8,*], Erica Ollmann Saphire[1,14,*,#]

[1]Center for Infectious Disease and Vaccine Discovery, La Jolla Institute for Immunology, La Jolla, CA, USA

[2]Division of Structural Studies, Medical Research Council Laboratory of Molecular Biology, Cambridge, UK

[3]Department of Cell and Virus Structure, Max Planck Institute of Biochemistry, Martinsried, Germany

[4]Influenza Research Institute, Department of Pathobiological Sciences, School of Veterinary Medicine, University of Wisconsin—Madison, Madison, WI, USA

[5]Integrated Research Facility at Fort Detrick, National Institute of Allergy and Infectious Diseases, National Institutes of Health, Fort Detrick, Frederick, MD, USA

[6]Microscopy Core, La Jolla Institute for Immunology, La Jolla, La Jolla, CA, USA

[7]Integral Molecular, Philadelphia, PA, USA

[8]Department of Microbiology and Immunology and Emory Vaccine Center, Atlanta, GA, USA

[9]Zalgen Labs LLC, Frederick, MD, USA

[10]Clinical Monitoring Research Program Directorate, Frederick National Laboratory for Cancer Research, Frederick, MD, USA

[11]Division of Virology, Institute of Medical Science, University of Tokyo, Tokyo, Japan

[12]The Research Center for Global Viral Diseases, National Center for Global Health and Medicine Research Institute, Tokyo, Japan

[13]Pandemic Preparedness, Infection and Advanced Research Center (UTOPIA), University of Tokyo, Tokyo, Japan

[14]Department of Medicine, University of California San Diego, La Jolla, CA, USA

[15]Current address: Department of Molecular Biosciences, the University of Texas at Austin, Austin, TX, USA

[16]Current address: Guangzhou Regenerative Medicine and Health—Guangdong Laboratory, Guangzhou Institutes of Biomedicine and Health, Chinese Academy of Sciences, Science Park, Guangzhou, Guangdong Province, China

[17]Current address: Arcturus Therapeutics, San Diego, CA, USA

[18]Current address: Eli Lilly, San Diego, CA, USA

[19]Current address: Department of Biochemistry, University of Toronto, Toronto, Canada

[20]Current address: Vall d'Hebron Institute of Oncology, Hospital del Mar Research Institute, Barcelona, Spain

[$]equal contributions

*Corresponding authors:

John A. G. Briggs, briggs@biochem.mpg.de

Gabriella Worwa, gabriella.worwa@nih.gov

Carl W. Davis, cwdavi2@emory.edu

Rafi Ahmed, rahmed@emory.edu

Erica Ollmann Saphire, erica@lji.org

[#] Lead Contact: Erica Ollmann Saphire; erica@lji.org

Supplementary Table 1. Structures of human mAb 3A6 in complex with the Ebola virus stalk–MPER peptide: Crystallographic statistics.

	**3A6**	**3A6-stalk–MPER**
**Data collection**		
Beamline	APS 231D-B	SSRL 12-2
Space group	*P*41	*P*1 21 1
Unit cell (a, b, c) (Å)	53.66, 65.67, 125.56	52.29, 66.42, 67.98
(α, β, γ) (°)	98.7, 91.4, 96.0	90, 104.2, 90
Resolution range (last shell) (Å)	43.51–2.50 (2.59–2.50)	65.89–1.27 (1.32–1.27)
Multiplicity	1.7 (1.7)	2.0 (1.8)
Completeness (spherical) (%)	-	93.92 (60.80)
Completeness (ellipsoidal) (%)	78.1 (10.1)	-
R_{meas} (%)		0.025 (0.36)
R_{pim} (%)		0.018 (0.25)
$CC_{1/2}$ (%)	0.987 (0.53)	0.999 (0.88)
Average I/σ(I)	2.7 (1.6)	11.0 (2.29)
**Refinement**		
R_{work}/R_{free}	0.192/0.249	0.167/0.179
Number of atoms		
Macromolecules	13179	3431
Solvent	215	639
Root-mean-square deviations		
Bond lengths (Å)	0.014	0.008
Bond angles (°)	1.67	1.02
Average B (A2)	39.44	19.54
Macromolecules	39.55	17.02
Solvent	32.9	33.09
Ramachandran plot		
Outliers (%)	0	0
Allowed (%)	2.54	1.38
Favored (%)	97.46	98.16
Protein Databank (PDB) ID	7RPT	7RPU

APS, advanced photon source; MPER, membrane proximal external region; SSRL, Stanford synchrotron radiation lightsource.

Supplementary Table 2. Residues D632 and P636 of the Ebola virus glycoprotein MPER are key for mAb 3A6 binding: Flow cytometric analysis of 3A6 binding to EBOV $GP_{1,2\Delta MLD}$ bearing the indicated alanine variant. Mean 3A6 binding to the indicated $GP_{1,2\Delta MLD}$ variants are expressed as percentage of cell-surface reactivity relative to wild-type EBOV $GP_{1,2\Delta MLD}$ with ranges (half of the maximum minus minimum values). Values shaded in orange indicate critical residues. Binding represents the average of two technical replicates.

$GP_{1,2\Delta MLD}$ variant	Mean	Range
**I627A**	94	16
**H628A**	34	10
**D629A**	70	6
**F630A**	71	7
**V631A**	173	11
**D632A**	10	1
**K633A**	84	11
**T634A**	86	13
**L635A**	103	1
**P636A**	14	5
**D637A**	105	5
**Q638A**	73	5
**G639A**	58	4

Supplementary Table 3. Residues D632 and P636 of the Ebola virus glycoprotein MPER are key for mAb 3A6 binding: Neutralization of P636S-bearing EbolaΔVP30-eGFP virions by multiple mAbs. $GP_{1,2}$ P636S-bearing "biologically-contained" EbolaΔVP30-eGFP was tested against a panel of mAbs identified by Davis, et al. [1]in a plaque reduction neutralization assay. Listed are mAbs having potent (orange highlight), partial (grey), and no (green) neutralization activity against this virus. Anti-EBOV $GP_{1,2}$ stalk–MPER-specific mAbs 9.6.3.A06 and 2.10.1 E06 are written in bold purple font. The number of plaques observed after 10 µg/mL of mAb treatment in two independent experiments is listed with the mean values in parentheses. MPER, membrane proximal external region, RBS, receptor binding site. [*]same short name for two distinct mAbs.

mAb	Short name	Antigenic site	# Virus plaques (mean)
-			168/162 (165)
**9.6.3 A06**	3A6	GP_2 MPER	124/138 (131)
**2.10.1 E06**	1E6	GP_2 MPER	126/124 (125)
9.6.3 D06	3D6	GP_1 glycan cap	97/109 (103)
2.1.1 D05	1D5	GP_1 core	0/0 (0)
5.1.7 D03	7D3	GP_1 core	10/9 (9.5)
9.6.1 A09	1A0	GP_1 core	7/7 (7)
9.20.1 A02	1A2*	GP_1 core	0/0 (0)
5.6.1 A02	1A2*	GP_2 fusion loop	0/0 (0)
9.6.3 A04	3A4	GP_2 fusion loop	0/0 (0)
5.24.12 C11	12C11	$GP_{1,2}$ trimer base	0/0 (0)
5.24.2 C06	2C6	$GP_{1,2}$ trimer base	0/0 (0)
9.20.1 C03	1C3	GP_1 RBS	0/0 (0)
5.24.2 A12	2A12	GP_1 RBS	0/0 (0)
5.1.10 B3	10B3	GP_1 core	55/72 (63.5)
5.1.13 G03	13G3	GP_2 fusion loop	20/16 (18)
2.1.1 D07	1D7	GP_1-GP_2 interface	50/61 (55.5)
2.1.7 G07	7G7	GP_1-GP_2 interface	32/31 (31.5)
5.24.2 C05	2C5	$GP_{1,2}$ trimer base	66/68 (67)
9.20.1 D09	1D9	$GP_{1,2}$ trimer base	59/49 (54)
5.24.2 D11	2D11	$GP_{1,2}$ trimer base	18/20 (19)

Supplementary Table 4. Binding of mAb 3A6 lifts Ebola virus glycoprotein relative to the membrane surface: Cryogenic electron tomography data acquisition and processing parameters.

**Sample**	**VLP-GP$_{1,2}$– 3A6 Fab EMD-45835**	**VLP-GP$_{1,2}$– KZ52 Fab EMD-45833**	**VLP-GP$_{1,2}$– KZ52 Fab EMD-45834**	**VLP-GP$_{1,2}$– 3A6-KZ52 Fab EMD-45836**	**VLP-GP$_{1,2}$– 3A6-KZ52 Fab EMD-45837**
Microscope	Titan Krios	Titan Krios	Titan Krios	Titan Krios	Titan Krios
Voltage (keV)	300	300	300	300	300
Energy filter (eV)	20	20	20	20	20
Detector	Gatan K3	Gatan K3	Gatan K3	Gatan K3	Gatan K3
Recording Mode	Counting	Counting	Counting	Counting	Counting
Pixel size (Å)	1.386	1.386	1.386	1.386	1.386
Defocus range (μm)	-2 to -4.5	-2 to -4.5	-2 to -4.5	-2 to -4.5	-2 to -4.5
Acquisition scheme	-60/60°, 3°	-60/60°, 3°	-60/60°, 3°	-60/60°, 3°	-60/60°, 3°
Total Dose (electrons/Å^2)	~120	~120	~120	~120	~120
Frame number	10	10	10	10	10
Tomograms	26	18	18	42	42
Subtomograms	9,602	13,520	13,520	40,072	40,072
Symmetry	C3	C3	C3	C3	C3
Resolution at 0.143 FSC (Å)	17.7	8.9	12.7	7.4	9.3

Fab, fragment antigen binding; FSC, Fourier shell correlation; GP, glycoprotein; GP$_{1,2}$, glycoprotein subunits 1 and 2; VLP, virion-like particle.

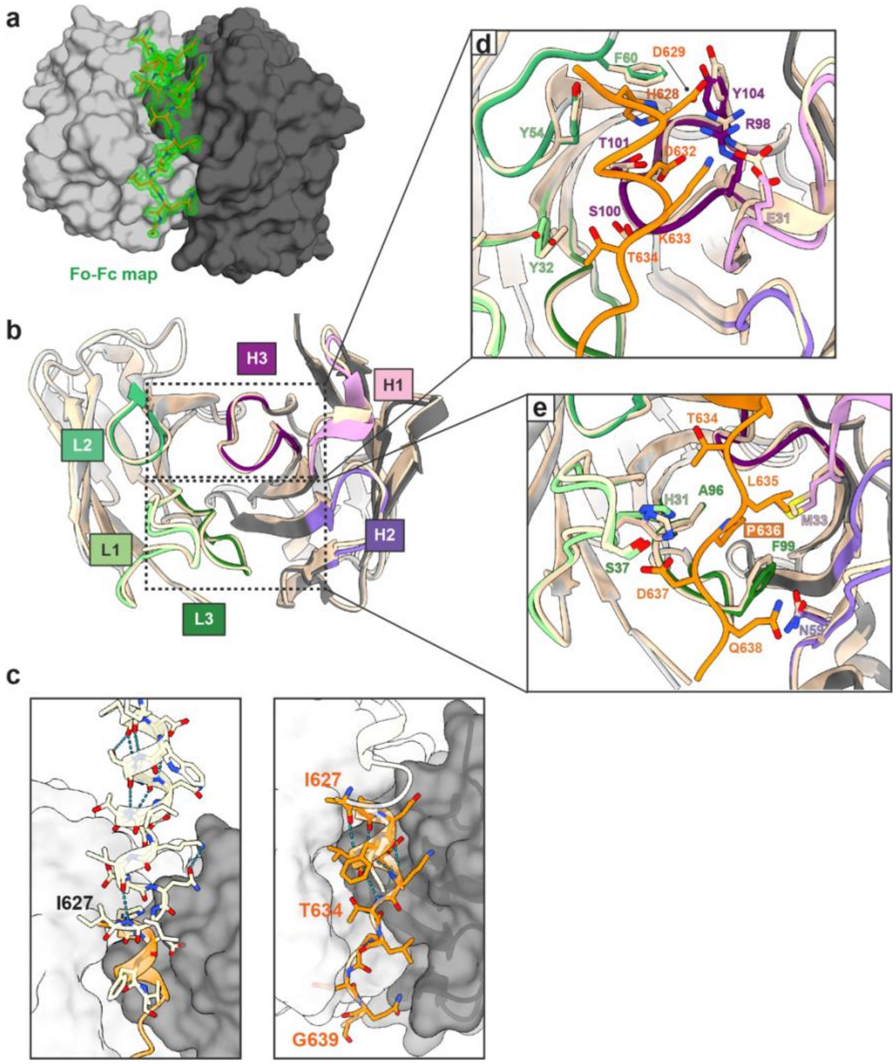

Supplementary Figure 1. Crystal structures of unbound human mAb 3A6 and mAb 3A6 bound to the Ebola virus glycoprotein stalk–MPER. (a) Fo–Fc density from a simulated annealing composite omit map (green mesh; 1.3 Å) calculated for the 3A6 stalk–MPER complex structure with the EBOV stalk–MPER peptide deleted from the model. **(b)** Crystal structure of the 3A6–peptide complex (colored by CDRs as in Figure 1C) superposed onto the 3A6 structure (tan), RMSD: 0.461 Å. **(c)** The 3A6-stalk–MPER structure (stalk–MPER peptide shown in orange, Fab in greys) docked onto the stalk region of the EBOV trimeric GP_2 ectodomain (yellow, PDB ID: 5JQ7) using the overlapping portion of the 3A6 epitope as a guide. The peptide representing GP_2 residues I627–G639 in the crystal structure extends the current observation of the stalk–MPER and shows that the α-helical structure continues until residue T634 and unravels thereafter. **(d and e**) Shifts and rotations of residue side chains in the CDRs upon 3A6 binding. P636, associated with escape from 3A6 after change P636S, is boxed in orange. CDR, complementarity determining region; EBOV, Ebola virus; $GP_{2,}$ glycoprotein subunit 2; MPER, membrane proximal external region; PDB, Protein Data Bank; RMSD, root-mean-square deviation.

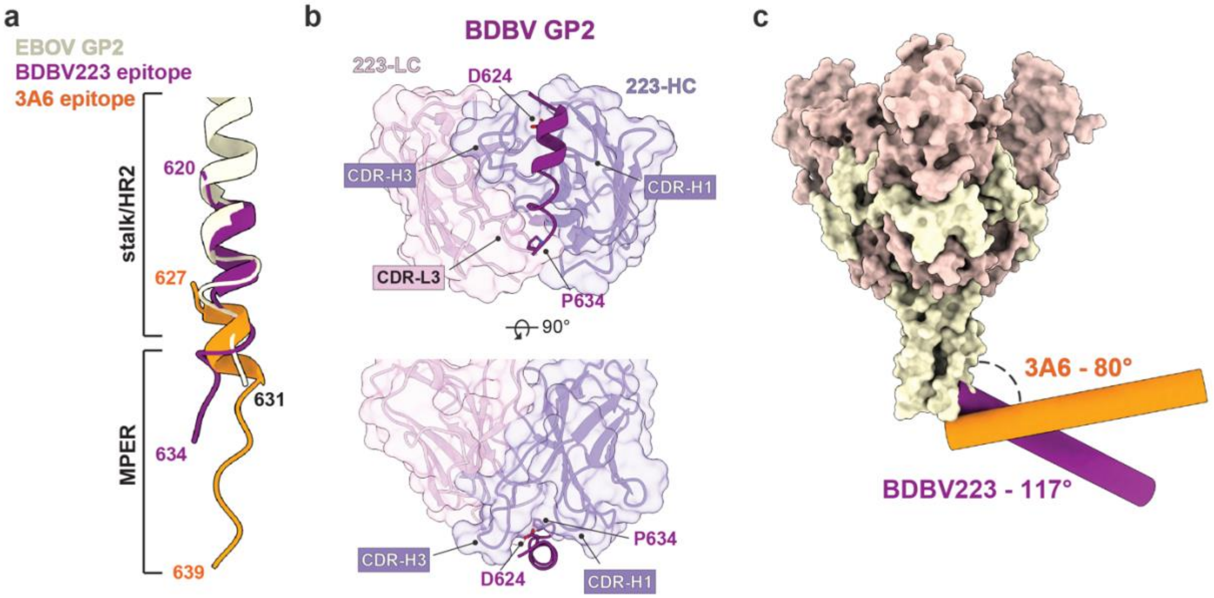

Supplementary Figure 2. Comparison of 3A6 to the stalk–MPER binding of mAb BDBV223. (a) Alignment of EBOV GP_2 HR2 (cream; top) and the peptide epitopes of BDBV223 (purple; middle) and 3A6 (orange; bottom). **(b)** BDBV223 contacts the stalk epitope primarily through CDR-H3. D624 and P634 form key contacts to the Fab. **(c)** BDBV223 and 3A6 approach EBOV $GP_{1,2}$ at different angles. BDBV, Bundibugyo virus; CDR, complementarity determining region; EBOV, Ebola virus; $GP_{2,}$ glycoprotein subunit 2; HC, heavy chain; HR2, heptad repeat region 2; LC, light chain; MPER, membrane proximal external region.

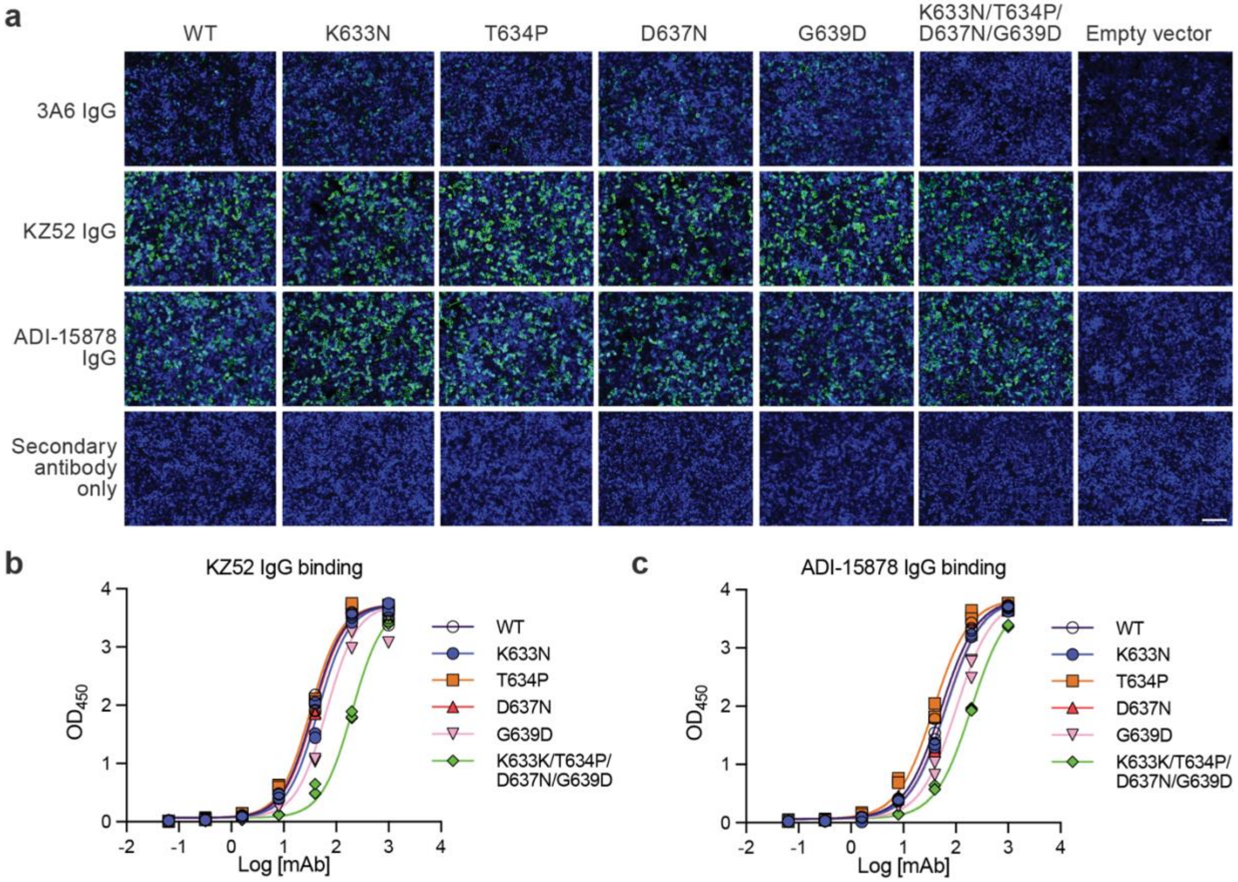

Supplementary Figure 3. Residues D632 and P636 of the Ebola virus glycoprotein MPER are key for mAb 3A6 binding. **(a)** Representative widefield fluorescent microscopy images used for quantification for Figure 2a (n=3 independent transfection experiments). Scale bar, 100 µm. ELISA measurement of conformational IgG mAbs KZ52 (**b**) and ADI-15878. **(c)** Binding to EBOV $GP_{1,2}$ WT or variants thereof (EBOV $GP_{1,2\Delta TM/\Delta MLD}$) containing the same amino acid residue changes as in Figure 2a. Symbols represent individual data points from n=3 technical replicates. $GP_{2,}$ glycoprotein subunit 2; EBOV, Ebola virus; ELISA, enzyme-linked immunosorbent assay; $GP_{2,}$ glycoprotein subunit 2; IgG, immunoglobulin G; mAb, monoclonal antibody; OD, optical density; WT, wild-type.

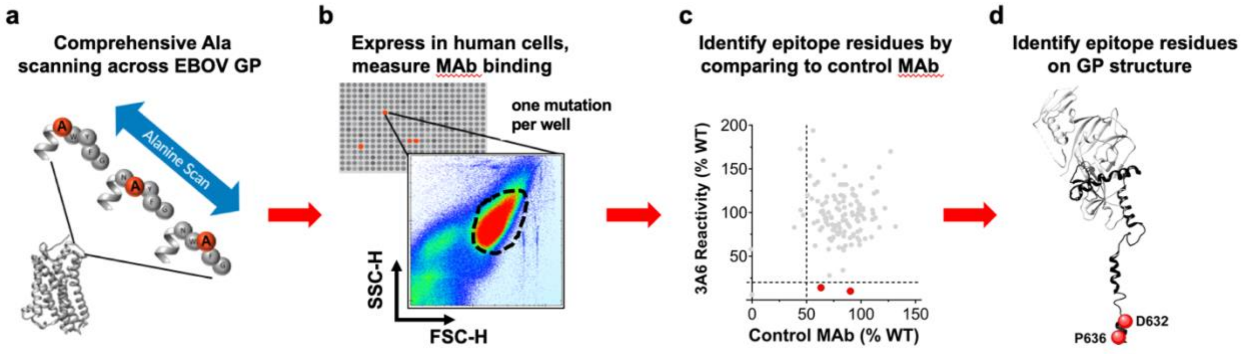

Supplementary Figure 4. Strategy for the identification of key residues for 3A6 binding. (**a**) An alanine scan library of EBOV $GP_{1,2\Delta MLD}$ was created and (**b**) expressed for 22 h in human cells in 384 well plates, one mutation per well. MAb was added to each well and binding detected by fluorescent secondary antibody. For each well, high-throughput flow cytometry was used to determine the mean fluorescence per cell, in a cell population identified by gating for side scatter and forward scatter. (**c**) The fluorescence values for each well were background subtracted and normalized to MAb binding with cells expressing WT $GP_{1,2\Delta MLD}$, enabling comparison of the test MAb's binding across the library to binding by other conformational 'control' MAbs. This identified critical mutations (red dots) resulting in low test MAb binding, but with high binding by a control MAb, validating $GP_{1,2\Delta MLD}$ expression and folding. (**d**) Critical epitope residues were mapped onto the $GP_{1,2}$ structure.

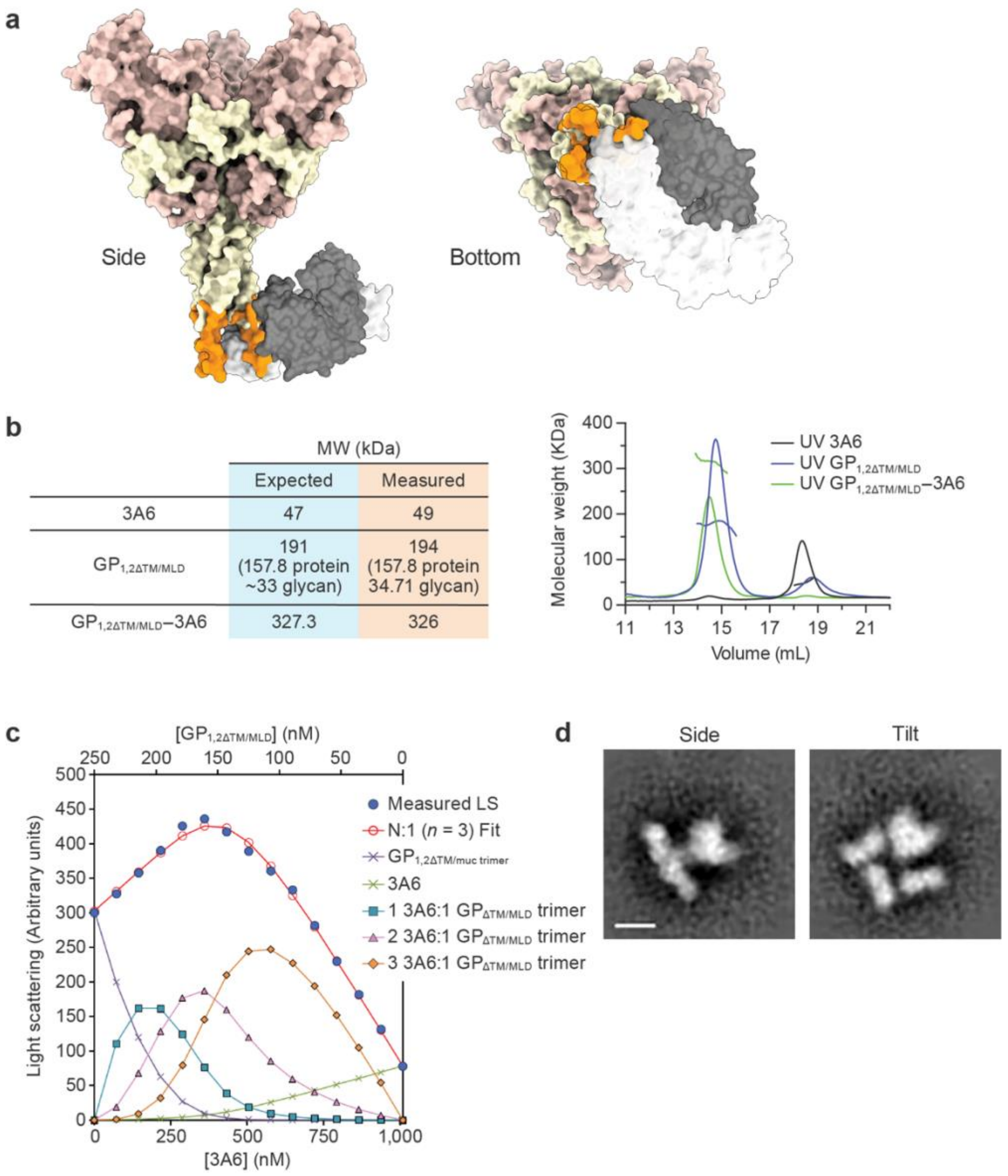

Supplementary Figure 5. Three copies of 3A6 bind to the EBOV $GP_{1,2}$ trimer. (a) A model of EBOV the $GP_{1,2}$ trimer stabilized by an exogenous trimerization domain (PDB 5JQ3) demonstrates Fab 3A6 binding is incompatible with HR2 constrained in a tight helical bundle. **(b)** SEC-MALS analysis of trimeric EBOV $GP_{1,2\Delta TM/\Delta MLD}$ and 3A6s separately and as a preformed complex. The measured molecular weights demonstrate an approximate 3 to 1 stoichiometry for 3A6 binding to the glycoprotein trimer. **(c)** CG-MALS analysis of trimeric $GP_{1,2\Delta TM/\Delta MLD}$–3A6. Light scattering intensity is shown as a function of composition, with blue circles indicating the measured light scattering intensity for each gradient plateau and red circles indicating the fit to a model of up to three 3A6s bound to one GP_2 trimer with equal affinity. The resulting fitted model calculates that three 3A6s bind to $GP_{1,2}$ with equal affinity of K_D = 52·15 (±1·3) nM. **(d)** Negative-stain electron microscopy analysis of $GP_{1,2}$ in complex with 3A6 shows full occupancy of 3A6. CG-MALS, composition gradient multi-angle light scattering; EBOV, Ebola virus; Fab, fragment antigen binding; $GP_{1,2}$, glycoprotein subunits 1 and 2; LS, light scattering; SEC-MALS, size-exclusion chromatography coupled to multi-angle light scattering.

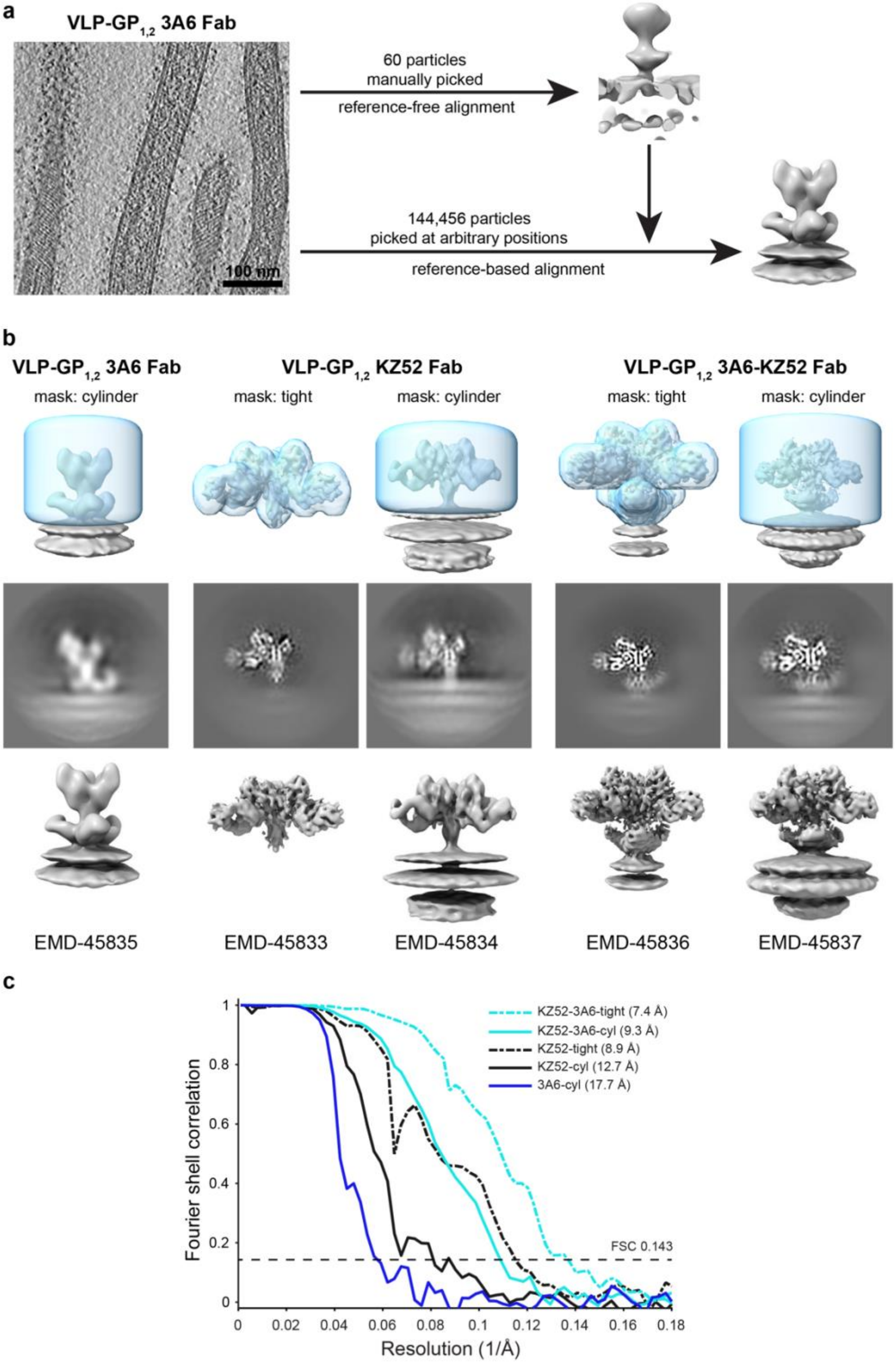

Supplementary Figure 6. Cryo-ET image processing workflow. (**a**) Cryo-ET data processing workflow illustrated for VLP-$GP_{1,2}$ 3A6 Fab as an example. A representative tomogram slice is shown. Sixty particles were manually picked and averaged, reference-free, to generate a first structure. This structure is used as a reference for iterations of reference-based alignment of 144,456 particles picked from arbitrary positions

along the membrane, leading to a final average from 9,602 particles. See also Supplementary Table 4. (**b**) Top row: the masks (light cyan) used for the alignment process are overlayed on the averages (gray). Middle row: central slices of the subtomogram averaged maps. Bottom row: 3D surface visualization of the final maps. (**c**). Fourier shell correlation for the reconstruction of GP1,2 in complex with the indicated antibody fragment. Graph generated by Matlab using relion postprocess.star files. FSC, Fourier shell correlation. Solid FSC curves are using the cylinder-shaped alignment mask (cyl), the dashed FSC curves are using the tight alignment mask (tight).

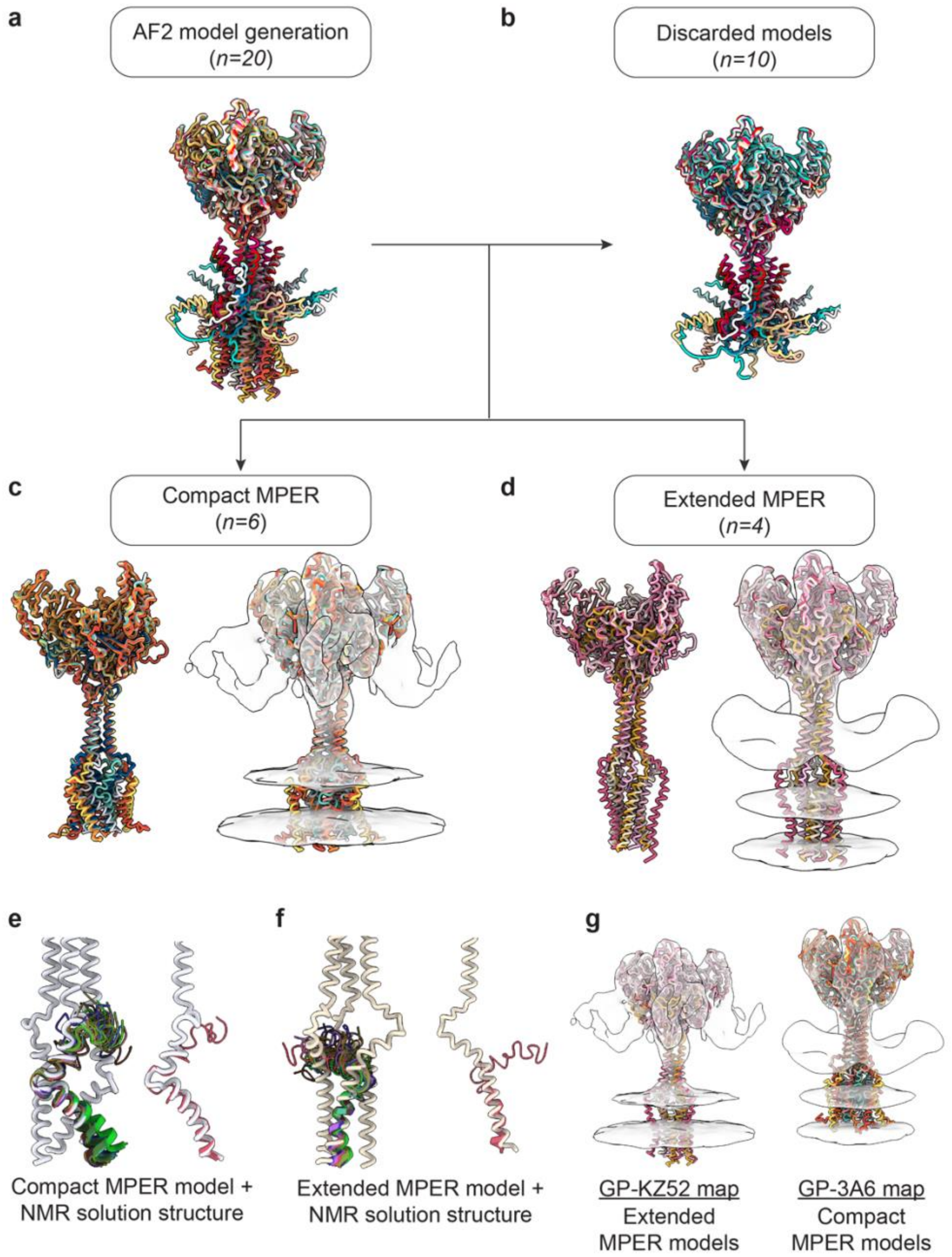

Supplementary Figure 7. Structural modeling of full-length EBOV $GP_{1,2}$. (**a**) AlphaFold 2 (AF2) [2-4] was used to generate 20 models of transmembrane (TM)-containing EBOV **$GP_{1,2}$**. (**b**) Models displaying the TM in an upward position were discarded. The remaining models were sorted into those with compact (**c**) or extended (**d**) MPER and TM regions. (**e**) Models with a compact MPER/TM resemble the EBOV MPER-TM solution structure (PDB 5T42), while those that have a more extended MPER are divergent. (**f**) Extended and compact models poorly fit the **$GP_{1,2}$**-KZ52 and **$GP_{1,2}$**-3A6 maps, respectively. AF2, AlphaFold 2; EBOV, Ebola virus; GP, glycoprotein; MPER, membrane proximal external region; NMR, nuclear magnetic resonance; TM, transmembrane [domain].

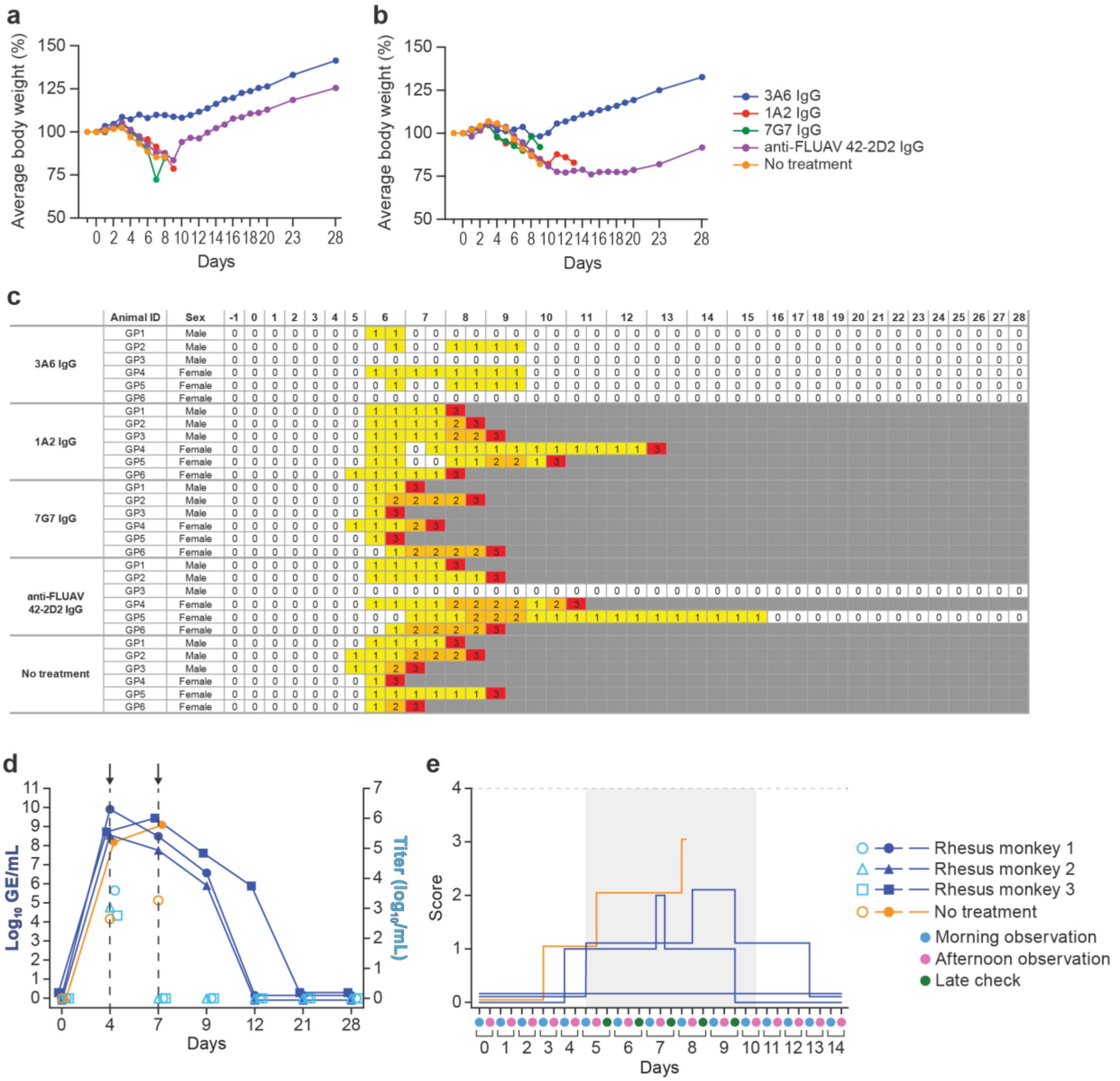

Supplementary Figure 8. Low-dose mAb 3A6 monotherapy protects domesticated guinea pigs and rhesus monkeys against EVD. Body weights of **(a)** male and **(b)** female domesticated guinea pigs and **(c)** their clinical scoring over the course of the experiment shown in Figure 5A. **(d)** Viremia as determined by RT-qPCR and **(e)** clinical scoring of rhesus monkeys over the course of the experiment shown in Figure 5B (critical period is highlighted in grey). FLUAV, influenza A virus; GE, genome equivalents; IgG, immunoglobulin G.

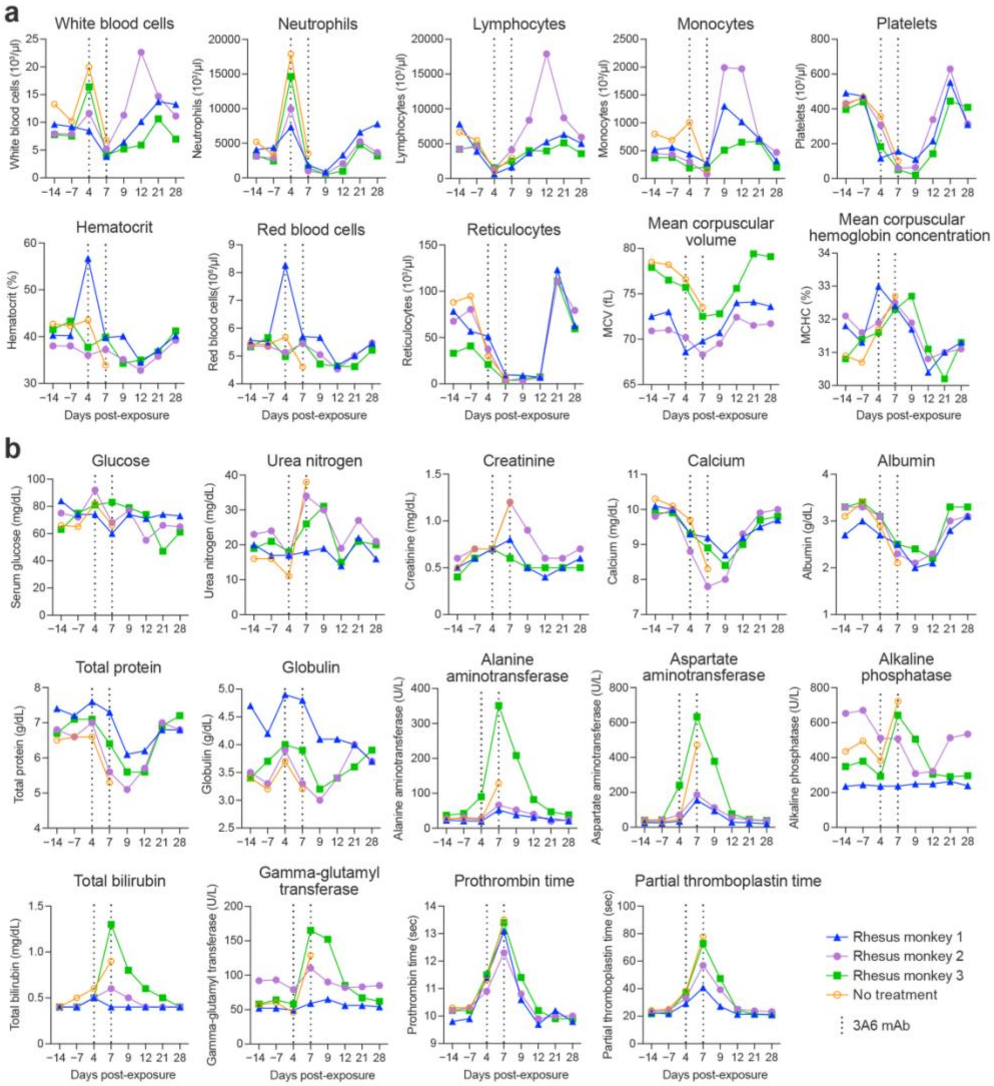

Supplementary Figure 9. Low-dose mAb 3A6 monotherapy protects rhesus monkeys against EVD. **(a)** Hematology and **(b**) blood chemistry for three rhesus monkeys over the course of the experiment shown in Figure 5B. The animals were infected with EBOV on Day 0. Black dotted lines indicate delivery of antibody treatments on Days 4 and 7 post-exposure.

REFERENCES

1. Davis, C.W., *et al.* Longitudinal analysis of the human B cell response to Ebola virus infection. *Cell* **177**, 1566-1582 e1517 (2019).
2. Jumper, J., *et al.* Highly accurate protein structure prediction with AlphaFold. *Nature* **596**, 583-589 (2021).
3. Evans, R., *et al.* Protein complex prediction with AlphaFold-Multimer. *bioRxiv*, 2021.2010.2004.463034 (2022).
4. Mirdita, M., *et al.* ColabFold: making protein folding accessible to all. *Nat Methods* **19**, 679-682 (2022).
