# Supplementary material for: Anti-Ebola virus mAb 3A6 protects highly viremic animals from fatal outcome via binding GP(1,2) in a position elevated from the virion membrane
Source: Nat Commun. 2025 Feb 3;16:1293. doi: 10.1038/s41467-025-56452-2 (PMC11791206; doi:10.1038/s41467-025-56452-2)
Supplement: Supplementary file 2 — Reporting Summary [file 41467_2025_56452_MOESM2_ESM.pdf]

nature portfolio

Corresponding author(s): Erica Ollmann Saphire

Last updated by author(s): 11/04/2024

Reporting Summary

Nature Portfolio wishes to improve the reproducibility of the work that we publish. This form provides structure for consistency and transparency in reporting. For further information on Nature Portfolio policies, see our Editorial Policies and the Editorial Policy Checklist.

Statistics

For all statistical analyses, confirm that the following items are present in the figure legend, table legend, main text, or Methods section.

n/a	Confirmed	
☐	☒	The exact sample size (*n*) for each experimental group/condition, given as a discrete number and unit of measurement
☐	☒	A statement on whether measurements were taken from distinct samples or whether the same sample was measured repeatedly
☒	☐	The statistical test(s) used AND whether they are one- or two-sided<br>*Only common tests should be described solely by name; describe more complex techniques in the Methods section.*
☒	☐	A description of all covariates tested
☒	☐	A description of any assumptions or corrections, such as tests of normality and adjustment for multiple comparisons
☒	☐	A full description of the statistical parameters including central tendency (e.g. means) or other basic estimates (e.g. regression coefficient) AND variation (e.g. standard deviation) or associated estimates of uncertainty (e.g. confidence intervals)
☒	☐	For null hypothesis testing, the test statistic (e.g. *F*, *t*, *r*) with confidence intervals, effect sizes, degrees of freedom and *P* value noted<br>*Give P values as exact values whenever suitable.*
☒	☐	For Bayesian analysis, information on the choice of priors and Markov chain Monte Carlo settings
☒	☐	For hierarchical and complex designs, identification of the appropriate level for tests and full reporting of outcomes
☒	☐	Estimates of effect sizes (e.g. Cohen's *d*, Pearson's *r*), indicating how they were calculated

Our web collection on statistics for biologists contains articles on many of the points above.

Software and code

Policy information about availability of computer code

Data collection	not applicable
Data analysis	not applicable

For manuscripts utilizing custom algorithms or software that are central to the research but not yet described in published literature, software must be made available to editors and reviewers. We strongly encourage code deposition in a community repository (e.g. GitHub). See the Nature Portfolio guidelines for submitting code & software for further information.

Data

Policy information about availability of data

All manuscripts must include a data availability statement. This statement should provide the following information, where applicable:

- Accession codes, unique identifiers, or web links for publicly available datasets
- A description of any restrictions on data availability
- For clinical datasets or third party data, please ensure that the statement adheres to our policy

Structure factors and associated model coordinates have been deposited in the Protein Data Bank (PDB; http://www.rcsb.org) under PDB accession codes 7RPU (3A6–stalk–MPER) and 7RPT (3A6 structure). Tomographic reconstructions have been deposited in the Electron Microscopy Data Bank (EMDB; https://www.ebi.ac.uk/emdb/) under accession codes EMD-45833, EMD-45834, EMD-45835, EMD-45836, and EMD-45837.

Research involving human participants, their data, or biological material

Policy information about studies with human participants or human data. See also policy information about sex, gender (identity/presentation), and sexual orientation and race, ethnicity and racism.

Reporting on sex and gender	not applicable
Reporting on race, ethnicity, or other socially relevant groupings	not applicable
Population characteristics	not applicable
Recruitment	not applicable
Ethics oversight	not applicable

Note that full information on the approval of the study protocol must also be provided in the manuscript.

Field-specific reporting

Please select the one below that is the best fit for your research. If you are not sure, read the appropriate sections before making your selection.

[x] Life sciences [ ] Behavioural & social sciences [ ] Ecological, evolutionary & environmental sciences

For a reference copy of the document with all sections, see nature.com/documents/nr-reporting-summary-flat.pdf

Life sciences study design

All studies must disclose on these points even when the disclosure is negative.

Sample size	No sample size calculations were performed. Sample sizes are based upon previous experimental groups used by us and others in the field.
Data exclusions	No data was excluded from the analysis.
Replication	Findings for animals studies were not reproduced. For other, non-animal studies, results were confirmed by at least one independent repeat, with no data excluded.
Randomization	Groups for animal studies were allocated randomly.
Blinding	Investigators were not blinded.

Reporting for specific materials, systems and methods

We require information from authors about some types of materials, experimental systems and methods used in many studies. Here, indicate whether each material, system or method listed is relevant to your study. If you are not sure if a list item applies to your research, read the appropriate section before selecting a response.

Materials & experimental systems

n/a	Involved in the study	
[ ]	[x]	Antibodies
[ ]	[x]	Eukaryotic cell lines
[x]	[ ]	Palaeontology and archaeology
[ ]	[x]	Animals and other organisms
[x]	[ ]	Clinical data
[x]	[ ]	Dual use research of concern
[x]	[ ]	Plants

Methods

n/a	Involved in the study	
[x]	[ ]	ChIP-seq
[ ]	[x]	Flow cytometry
[x]	[ ]	MRI-based neuroimaging

Antibodies

Antibodies used	anti-EBOV antibodies 3A6, KZ52, and ADI-15878 were produced in-ho<br>anti-human IgG-alexa Fluor 488-conjugated secondary antibody (Thermo Fisher Scientific).<br>DyLight 488 anti-human IgG secondary antibody (SA5-10126; Thermo Fisher Scientific)<br>HRP-goat anti-human IgG (#109036006; Thermo Fisher Scientific)

Validation	Anti-Ebola antibodies were validated for binding to Ebolavirus glycoprotein via ELISA and flow cytometry. Binding sites for each mAb have been structurally defined.<br>Commercial antibodies are validated "by Relative expression to ensure that the antibody binds to the antigen stated." Further, a certificate of analysis is available for each antibody.

Eukaryotic cell lines

Policy information about cell lines and Sex and Gender in Research

Cell line source(s)	HEK 293T cells were obtained from ATCC. Vero cells were obtained from ATCC and were used to create stable cell lines expressing VP30. Expi293 cells used for antibody production and Drosophila Schneider 2 (S2) cells used for glycoprotein expression were obtained from Thermo Fisher Scientific.
Authentication	Cell lines were not authenticated
Mycoplasma contamination	All cell lines tested negative for mycoplasma.
Commonly misidentified lines (See ICLAC register)	HEK293T cells are regularly used in our lab due to the high transfections rate.

Animals and other research organisms

Policy information about studies involving animals; ARRIVE guidelines recommended for reporting animal research, and Sex and Gender in Research

Laboratory animals	Hartley strain domesticated guinea pigs (Cavia porcellus (Linnaeus, 1758)) and rhesus monkeys (Macaca mulatta (Zimmermann, 1780)) were used in these studies.
Wild animals	This study did not involve wild animals.
Reporting on sex	Equal numbers of both sexes for each animal model were used. Only aggregated data are presented as no sex-based differences were observed
Field-collected samples	This study did not involve field-collected samples.
Ethics oversight	Animal exposure and treatment experiments using infectious EBOV were performed in the biosafety level 4 (BSL-4) laboratory at the Integrated Research Facility at Fort Detrick (IRF-Frederick), Division of Clinical Research (DCR), National Institutes for Allergy and Infectious Diseases (NIAID), National Institutes of Health (NIH) under accreditation (000777) by the Association for Assessment and Accreditation of Laboratory Animal Care (AAALAC), Laboratory Animal Welfare approval (D16-00602) by the Public Health Service (PHS), and United States Department of Agriculture (USDA) registration (51-F-0016). Animal experiments were approved by the NIAID DCR Animal Care and Use Committee (ACUC) and followed the recommendations provided in the Guide for the Care and Use of Laboratory Animals.

Note that full information on the approval of the study protocol must also be provided in the manuscript.

Plants

Seed stocks	This study did not involve plants.
Novel plant genotypes	*Describe the methods by which all novel plant genotypes were produced. This includes those generated by transgenic approaches, gene editing, chemical/radiation-based mutagenesis and hybridization. For transgenic lines, describe the transformation method, the number of independent lines analyzed and the generation upon which experiments were performed. For gene-edited lines, describe the editor used, the endogenous sequence targeted for editing, the targeting guide RNA sequence (if applicable) and how the editor was applied.*
Authentication	*Describe any authentication procedures for each seed stock used or novel genotype generated. Describe any experiments used to assess the effect of a mutation and, where applicable, how potential secondary effects (e.g. second site T-DNA insertions, mosiacism, off-target gene editing) were examined.*

Flow Cytometry

Plots

Confirm that:

- [ ] The axis labels state the marker and fluorochrome used (e.g. CD4-FITC).
- [ ] The axis scales are clearly visible. Include numbers along axes only for bottom left plot of group (a 'group' is an analysis of identical markers).
- [ ] All plots are contour plots with outliers or pseudocolor plots.
- [ ] A numerical value for number of cells or percentage (with statistics) is provided.

Methodology

Sample preparation	Plasmid clones were individually arrayed into 384-well plates and transfected into HEK 293T cells. Protein variants were cell-surface-expressed for 22 h. Primary antibodies were incubated with cells for 1 hour prior to the addition of an anti-human alexa Fluor 488-conjugated secondary antibody.
Instrument	Intellicyt
Software	Intellicyt software
Cell population abundance	n/a
Gating strategy	Antibody binding was assessed by detection of cellular fluorescence with a high-throughput flow cytometer (Intellicyt, Albuquerque, NM, USA). Background fluorescence was measured in vector-transfected control cells and mAb reactivity against the variants was calculated with respect to reactivity with GP1,2ΔMLD by subtracting the signal from mock-transfected controls and normalized to signals from WT GP1,2ΔMLD-transfected controls.

- [x] Tick this box to confirm that a figure exemplifying the gating strategy is provided in the Supplementary Information.
